# COVID-19 responses and coping in young Malaysians from low-income families

**DOI:** 10.3389/fpsyt.2023.1165023

**Published:** 2023-05-15

**Authors:** Li Ping Wong, Nik Daliana Nik Farid, Haridah Alias, Sofia Md Yusop, Zuhrah Musa, Zhijian Hu, Yulan Lin

**Affiliations:** ^1^Centre for Epidemiology and Evidence-Based Practice, Department of Social and Preventive Medicine, Faculty of Medicine, Universiti Malaya, Kuala Lumpur, Malaysia; ^2^Department of Epidemiology and Health Statistics, School of Public Health, Fujian Medical University, Fuzhou, China; ^3^Centre for Population Health, Department of Social and Preventive Medicine, Faculty of Medicine, Universiti Malaya, Kuala Lumpur, Malaysia; ^4^National Population and Family Development Board [Lembaga Penduduk dan Pembangunan Keluarga Negara (LPPKN)], Kuala Lumpur, Malaysia

**Keywords:** BRCS, DASS-21, parent-youth conflict, pandemic, PEQ

## Abstract

**Introduction:**

This study aimed to shed light on how young people from low-income families were responding to COVID-19.

**Methods:**

This cross-sectional study recruited young people aged between 18 and 24 years from the low-income-group communities. A convenience sampling approach was used. Google Surveys were used to gather data from the survey. The questionnaire consisted of an assessment of demographic characteristics, lifestyle factors, parent–youth conflict (Parental Environment Questionnaire, PEQ), resilient coping (Brief Resilient Coping Scale, BRCS), and psychological distress (Depression, Anxiety, and Stress Scale-short form, DASS-21).

**Results:**

A total of 561 complete responses were received. The results showed a low level of parent–child conflict in the overall study population, with a median PEQ of 48.0 [interquartile range (IQR) 36–48]. Higher parent–child conflicts were found in females than in males (OR = 1.75, 95% CI 1.19–2.57) and in youth from households with an income below MYR 2000 than those earning MYR 3,001–5,000 (OR = 4.39, 95% CI 2.40–8.03). A low prevalence of depression (12.5%), anxiety (15.2%), and stress (6.4%) was found. Parent–child conflict remains the strongest significant predictor for higher levels of depression (OR = 10.90, 95% CI 4.31–27.57), anxiety (OR = 11.92, 95% CI 5.05–28.14), and stress (OR = 4.79, 95% CI 1.41–16.33) symptoms. Poor resilient coping was the second strongest predictor for depression and anxiety symptoms. Regarding lifestyle factors, a lower level of physical exercise was associated with higher symptoms of depression. By demographics, females reported more severe symptoms of depression and anxiety than males. Young people from low-income households reported greater severity in symptoms of depression, anxiety, and stress than those from high-income households. Young people who are employed also reported greater severity of anxiety symptoms than those who are unemployed.

**Discussion:**

The COVID-19 pandemic continues to have an unpredictable impact on the lives of vulnerable youth in low-income families that warrants attention in future advocacy efforts.

## Introduction

The novel coronavirus that caused COVID-19 has caused tremendous adverse events in the economic, health, and social wellbeing of people worldwide (1). People of different ages, however, are impacted differently in all aspects of life; in particular, the pandemic poses considerable risks to and long-lasting impacts on adolescents and young people in various aspects with regard to employment, education, and mental wellbeing (2). Young people, especially vulnerable youth and those from low social classes, have been more heavily affected and report a strong impact of the COVID-19 crisis (3).

Parents, too, faced unique challenges during the pandemic, including fear and uncertainty of the health risks, in addition to stress from mobility constraints, isolation measures, working from home, financial impact, and the closure of schools and child-care facilities (4, 5). A large European study reported that parents experienced deteriorating wellbeing associated with home-schooling (6). Prominent evidence of deterioration in parents and their child's mental and behavioral health during the first month of the pandemic was also reported (7, 8). Collectively, pandemic-related stressors experienced by parents and their children have had negative implications on family relationships (9). Frequent negative parent–child interactions and conflicts with their children have been reported during the pandemic (10, 11). It has been noted that during the pandemic, sustaining mental health problems and family conflict is important to promote family members to practice healthy behaviors recommended by public health authorities (8). Undoubtedly, there is a growing concern about the psychological manifestations of the pandemic and parent–child wellbeing. The pandemic has triggered an array of psychological and family issues but the major concern is whether the pandemic-related stressors experienced remain after the pandemic and continue to pose a major threat to family harmony. Emergent symptoms of post-traumatic stress disorder (PTSD) in the context of the COVID-19 pandemic have been a concern, while some researchers have warned of a “second pandemic” of PTSD in the wake of the damage caused by this pandemic (12).

In Malaysia, several cross-sectional surveys point to significant declines in psychological and mental health among the general public (13–15); however, the impact on young people and particularly family relationships during the pandemic was relatively understudied. With the pandemic broadly under control, Malaysia started relaxing COVID-19 restrictions at the beginning of May 2022. Little is known whether the pandemic has lasting implications for young people in Malaysia. To fill this gap, the main aim of the present study was to explore the wellbeing of young people from low-income communities, by exploring their current state of parent–child conflict, resilient coping, and psychological distress. Specifically, we explore parent–youth conflict, coping, and healthy lifestyles in the prediction of psychological distress. This is particularly relevant as the pandemic lockdown has eased and normal life has resumed. We hypothesized that pandemic lockdowns and restrictions may create stressful conflict between young people and their parents, leading to greater psychological distress. In contrast, higher personal resilient coping attenuated the negative effect of conflict on their psychological stress levels. The findings of this study could be invaluable for mitigating the long-term consequences facing young people and their parents, as well as identifying recommendations that can be utilized in the case of any future pandemics.

## Methods

### Participants

The sample of young people was recruited from residents in the People Housing Project also known as the Program Perumahan Rakyat (PPR), a government settlement program for people from the low-income group (the Bottom 40% of the Malaysian household income or B40), in the state of Selangor and the Federal Territory of Kuala Lumpur, Malaysia. Malaysian households are classified into three income groups: Bottom 40% (B40), Middle 40% (M40), and Top 20% (T20). The B40 groups comprised 2.91 million households, and their monthly income is < RM4,850 (USD 1,099) (16). The average household income of the B40 group is MYR 3,172 (USD 718) (16). M40 is a group of households with a monthly income between RM4,850 and 10,959 (USD 1,099–2,483), whereas the T20 monthly income is over RM10,960 (USD 2,483) (16).

Field enumerators were trained to recruit eligible participants and assist them in answering the survey questions. A convenience sampling approach based on a “random walk” door-to-door recruitment strategy was used. Google Surveys were used to gather data from the survey. Participants who completed the surveys were assisted by field enumerators. Participants were also asked to refer their peers to take part in the study. Inclusion criteria were young people staying with their parents in the PPR and aged between 18 and 24 years. The questionnaire (Appendix 1) consisted of an assessment of demographic characteristics, lifestyle factors (smoking, alcohol consumption, exercise, healthy diet, and sleep quality), parent–child conflict, resilient coping, and psychological distress. The questionnaire is developed in English and translated into Malaysia's national language. The standard back-translation method was used, whereas the translated text was re-translated back into English by an independent translator. Both the English and Malaysia's national language versions of the questionnaire have also gone through content validation by experts and were subsequently pilot tested before conducting the survey.

The sample size was calculated using the online Raosoft sample size calculator (17). With an estimate of a response distribution of 50%, a confidence level of 95%, a margin error of 5%, and an estimate of a total of 39,000 B40 households in the state of Selangor and the Federal Territory of Kuala Lumpur (18, 19), the required sample size was 381. The study was conducted between May and August 2022. A total of 561 complete responses were received, which is 1.5 times larger than the estimated sample size.

### Assessment of parent–child conflict

The Parental Environment Questionnaire (PEQ) (20) was administered to tap perceptions of the parent–child relationship in the present study. The PEQ consists of 12 items assessing aspects of their relationships on a 4-point scale (1 = *definitely true*, 4 = *definitely false*). The score ranged from 12 to 48, all 12 items were summed, and higher overall scores reflected lower parent–child conflict. To the best of our knowledge, parent–child conflict has never been assessed in the Malaysian population. Cronbach's α value for Malaysia's national language version of PEQ in this study was 0.982, suggesting that the measure has a high level of internal consistency.

###  Assessment of resilient coping

The Brief Resilient Coping Scale (BRCS) is a 4-item measure designed to capture tendencies to cope with stress using a 5-point Likert scale “from ‘1' = *describes me not at all* to ‘5' = *describes me very well”* (21). Total sum scores range from 4 to 20, with a higher score implying higher resilient coping. Scores of 4–13 indicate low resilient coping, 14–16 indicate medium resilient coping, and 17–20 indicate high resilient coping (21). Cronbach's α value for the BRCS scale in this study was 0.941, suggesting that the measure has a high level of internal consistency.

###  Assessment of psychological distress

Psychological distress was measured using the Depression, Anxiety, and Stress Scale-short form (DASS-21) (22). Scores on three subscales— namely Depression (DASS-21-D), Anxiety (DASS-21-A), and Stress (DASS-21-S)—were generated. There are seven items in each subscale; the score of each subscale ranges from 0 to 21, with higher scores indicative of more severe symptoms of depression, anxiety, and/or stress. The cutoffs for depression (moderate 14–20, severe 21–27, and extremely severe ≥ 28), anxiety (moderate 10–14, severe 15–19, and extremely severe ≥ 20), and stress (moderate 19–25, severe 26–33, and extremely severe ≥ 34) were calculated (23) Cronbach's α value for the subscales DASS-21-D, DASS-21-A, and DASS-21-S in this study was 0.958, 0.944, and 0.952, respectively. This indicates that the DASS-21 scale used in our study population is a reliable psychometric instrument. The DASS-21 translated to Malaysia's national language used in a former study in Malaysia reported Cronbach's alpha values of 0.956 for the overall scale, 0.927 for the DASS-21-D, 0.865 for the DASS-21-A, and 0.882 for the DASS-21-S (14).

###  Statistical analyses

Descriptive statistics were computed on the dependent and independent variables. Frequency tables, charts, and proportions were used for data summarization. The proportion and its respective 95% confidence interval (CI) were calculated. We checked the assumption of normality, and parametric tests are used if the data follow a normal distribution; otherwise, non-parametric methods are used to compare groups. We ran univariate analyses followed by multivariable logistic regression analysis, including all factors showing significance (*p* < 0.05), to determine predictive factors of the three dimensions of the psychological distress of the DASS-21. Odds ratios (ORs), 95% confidence intervals (95% CIs), and *p*-values were calculated for each independent variable. Only significant factors in the univariate analyses, with a *p*-value of < 0.05, were selected for the multivariable regression analysis. The model fit of the multivariable logistic regression analysis was assessed using the Hosmer–Lemeshow goodness-of-fit test (24). All statistical analyses were performed using the Statistical Package for the Social Sciences version 20.0 (IBM Corp., Armonk, NY, USA). A *p*-value of < 0.05 was considered statistically significant.

###  Ethics considerations

This study was approved by the University of Malaya Research Ethics Committee (UM.TNC2/UMREC−1579). Participants were informed that their participation was voluntary. To consent to participate, participants were required to click “Yes, I consented to participate in this study”. The privacy of the participants and confidentiality of the data obtained were maintained. The availability of counseling services was made known to the study participants, and contact information was also provided to participants who need counseling or mental health services. Nevertheless, none of the participants reported severe psychological distress and reached out to the counseling services provided.

## Results

###  Sociodemographics and lifestyle

A total of 561 complete responses were received. The complete participants' demographics are shown in the first and second columns of Table 1. The majority of the study participants were aged between 18 and 21 years (69.7%). There was an almost equal amount of male and female participants. Nearly two-thirds (63.6%) reported a household family income of MYR 2,001–3,000. As shown in Table 1, only a minority reported ever smoking (17.3%) and consuming alcohol (12.1%). Over two-thirds (34.8%) reported often practicing healthy eating and doing physical exercises (37.6%) in the past 3 months.

**Table 1:** Factors associated with psychological distress.

	**Overall**	**Depression**			**Anxiety**			**Stress**		
		**Univariable analysis**		**Multivariable analysis**	**Univariable analysis**		**Multivariable analysis**	**Univariable analysis**		**Multivariable analysis**
		**Mild/moderate/severe/ extremely severe (*****n*** = **70)**		**Mild/moderate/severe/extremely severe vs. Normal** ^a^	**Mild/moderate/severe/extremely severe (*****n*** = **86)**		**Mild/moderate/severe/extremely severe vs. Normal** ^b^	**Mild/moderate/severe/extremely severe (*****n*** = **36)**		**Mild/moderate/severe/extremely severe vs. normal** ^c^
	***N*** **(%)**	***n*** **(%)**	***p*** **-value**	**OR (95% CI)**	***n*** **(%)**	***p*** **-value**	**OR (95% CI)**	***n*** **(%)**	***p*** **-value**	**OR (95% CI)**
**SOCIO DEMOGRAPHIC CHARACTERISTICS**										
**Age group (years)**										
18–21	391 (69.7)	49 (12.5)	1.000		53 (13.6)	0.097		29 (7.4)	0.189	
22–24	170 (30.3)	21 (12.4)			33 (19.4)			7 (4.1)		
**Gender**										
Male	281 (50.1)	16 (5.7)	*p* < 0.001	Ref	22 (7.8)	*p* < 0.001	Ref	4 (1.4)	*p* < 0.001	Ref
Female	280 (49.9)	54 (19.3)		3.11 (1.35–7.19)^**^	64 (22.9)		4.79 (2.10–10.91)^***^	32 (11.4)		3.37 (0.98–11.63)
**Occupation status**										
Student	354 (63.1)	47 (13.3)	0.352		49 (13.8)	0.013	1.01 (0.27–3.74)	28 (7.9)	0.154	
Employed	137 (24.4)	18 (13.1)			31 (22.6)		4.29 (1.05–17.46)^*^	6 (4.4)		
Unemployed	70 (12.5)	5 (7.1)			6 (8.6)		Ref	2 (2.9)		
**Average monthly household income (MYR)** ^¶^										
2,000 and below	100 (17.8)	51 (51.0)	*p* < 0.001	5.93 (2.23–15.75)^***^	59 (59.0)	*p* < 0.001	6.50 (2.59–16.27)^***^	29 (29.0)	*p* < 0.001	4.18 (1.30–13.51)^*^
2,001–3,000	357 (63.6)	10 (2.8)		0.51 (0.18–1.49)	16 (4.5)		0.57 (0.22–1.51)	2 (0.6)		0.14 (0.03–0.81)^*^
3,001–5,000	104 (18.5)	9 (8.7)		Ref	11 (10.6)		Ref	5 (4.8)		Ref
**Residence area**										
Urban	484 (86.3)	57 (11.8)	0.199		69 (14.3)	0.088		27 (5.6)	0.074	
Sub-urban	77 (13.7)	13 (16.9)			12 (22.1)			9 (11.7)		
**LIFESTYLE**										
**Smoking status in the past 3 months**										
Never smoke	464 (82.7)	61 (13.1)	0.398		76 (16.4)	0.163		35 (7.5)	0.012	4.34 (0.48–39.11)
Ever smoke	97 (17.3)	9 (9.3)			10 (10.3)			1 (1.0)		Ref
**Alcohol intake in the past 3 months**										
Never drink alcohol	493 (87.9)	67 (13.6)	0.031	1.02 (0.26–4.10)	82 (16.6)	0.019	1.03 (0.29–3.62)	35 (7.1)	0.108	
Ever drink alcohol	68 (12.1)	3 (4.4)		Ref	4 (5.9)		Ref	1 (1.5)		
**Doing physical exercises in the past 3 months**										
Never/seldom	137 (24.4)	44 (32.1)	*p* < 0.001	7.49 (1.46–38.49)^*^	50 (36.5)	*p* < 0.001	2.44 (0.59–10.09)	23 (16.8)	*p* < 0.001	4.14 (0.42–41.28)
Sometimes	213 (38.0)	20 (9.4)		5.13 (1.02–25.78)^*^	27 (12.7)		2.33 (0.58–9.28)	11 (5.2)		4.32 (0.43–43.22)
Often	211 (37.6)	6 (2.8)		Ref	9 (4.3)		Ref	2 (0.9)		Ref
**Practicing healthy eating in the past 3 months**										
Never/seldom	143 (25.5)	39 (27.3)	*p* < 0.001	0.39 (0.06–2.32)	46 (32.2)	*p* < 0.001	1.15 (0.22–6.03)	21 (14.7)	*p* < 0.001	0.81 (0.06–10.32)
Sometimes	223 (39.8)	25 (11.2)		0.27 (0.05–1.52)	34 (15.2)		0.91 (0.18–4.58)	13 (5.8)		0.52 (0.04–6.19)
Often	195 (34.8)	6 (3.1)		Ref	6 (3.1)		Ref	2 (1.0)		Ref
**Have enough sleep in a week in the past 3 months**										
Never/seldom	124 (22.1)	36 (29.0)	*p* < 0.001	0.80 (0.26–2.46)	46 (37.1)	*p* < 0.001	0.92 (0.32–2.65)	18 (14.5)	*p* < 0.001	0.42 (0.11–1.57)
Sometimes	208 (37.1)	22 (10.6)		1.01 (0.33–3.03)	26 (12.5)		0.74 (0.26–2.08)	11 (5.3)		0.57 (0.16–2.05)
Often	229 (40.8)	12 (5.2)		Ref	14 (6.1)		Ref	7 (3.1)		Ref
**Parent-child conflict**										
**Total Parental Environment Questionnaire (PEQ) score**										
Low score, High conflict (12–47)	203 (36.2)	63 (31.0)	*p* < 0.001	10.90 (4.31–27.57)^***^	75 (36.9)	*p* < 0.001	11.92 (5.05–28.14)^***^	32 (15.8)	*p* < 0.001	4.79 (1.41–16.33)^*^
High score, low conflict (48)	358 (63.8)	7 (2.0)		Ref	11 (3.1)		Ref	4 (1.1)		Ref
**Coping**										
**Total Brief Resilient Coping Scale (BRCS) score**										
Low resilient coping (4–13)	98 (17.5)	32 (32.7)	*p* < 0.001	9.16 (3.34–25.11)^***^	40 (40.8)	*p* < 0.001	6.63 (2.68–16.43)^***^	12 (12.2)	0.028	1.85 (0.60–5.67)
Medium resilient coping (14–16)	339 (60.4)	28 (8.3)		3.64 (1.40–9.48)^**^	30 (8.8)		2.42 (1.01–5.79)^*^	16 (4.7)		1.59 (0.55–4.54)
High resilient coping (17–20)	124 (22.1)	10 (8.1)		Ref	16 (12.9)		Ref	8 (6.5)		Ref

^*^p < 0.05. ^**^p < 0.01. ^***^p < 0.001.

^a^Hosmer–Lemeshow test, chi-square: 7.796, *p*-value: 0.454; Nagelkerke *R*^2^: 0.580.

^b^Hosmer–Lemeshow test, chi-square: 2.631, *p*-value: 0.955; Nagelkerke *R*^2^: 0.602.

^c^Hosmer–Lemeshow test, chi-square: 11.848, *p*-value: 0.158; Nagelkerke *R*^2^: 0.477.

^¶^1.USD = 4.41 MYR.

### 3.2. Parent–child conflict

**Figure 1:**
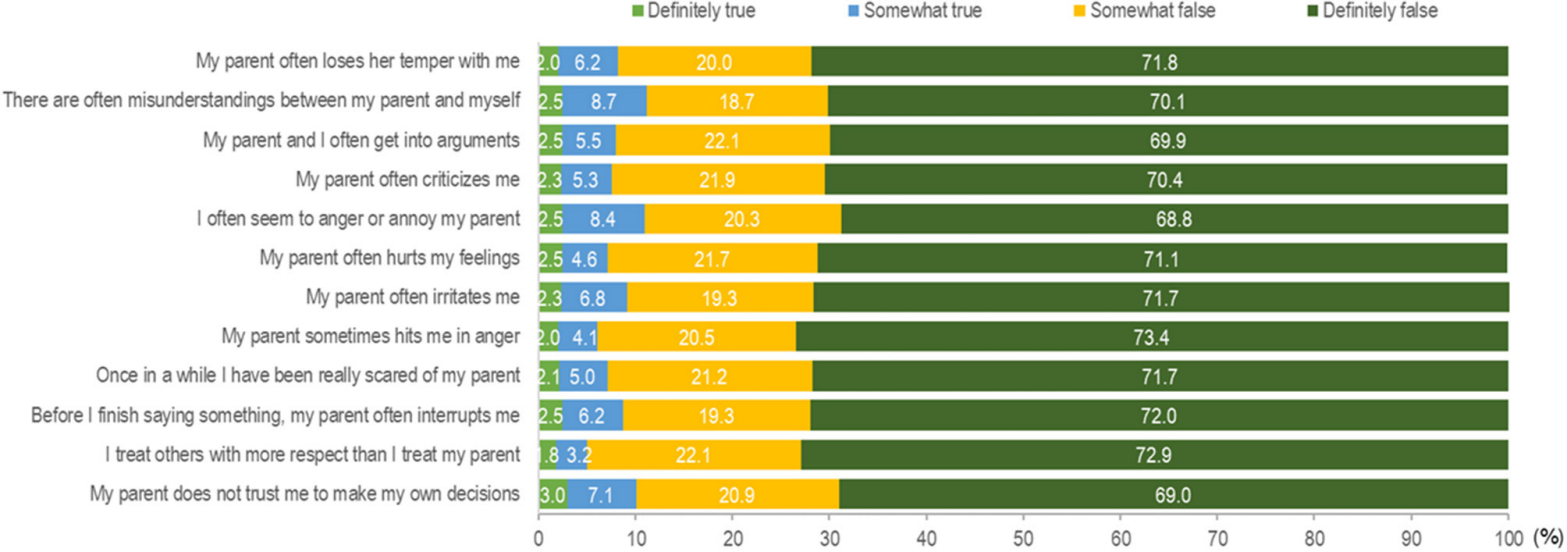
Responses for items in Parental Environment Questionnaire (PEQ).

Figure 1 shows the distribution of responses for the PEQ items. A total of 11.2% reported that there are often misunderstandings with their parents, followed by 10.9% who reported they often seem to annoy their parents, and 10.9% reported that their parents do not trust them to make their own decisions. The total PEQ score of the study participant ranged from 12 to 48, and the median PEQ score was 48.0 [interquartile range (IQR) 36–48]. The PEQ score was categorized as 12–47 or 48, based on the median split; as such, a total of 203 (36.2%, 95% CI 32.2–40.3) were categorized as having a score of 12–47, and 358 (63.8%, 95% CI 59.7–67.8) were categorized as having a score of 48. As shown in Appendix 2, by demographics, the odds of lower PEQ scores were more prominent among females than males (OR = 1.75, 95% CI 1.19–2.57) and in youth from households with an income below MYR 2,000 than those earning MYR 3,001–5,000 (OR= 4.39, 95% CI 2.40–8.03).

###  Resilient coping

**Figure 2:**
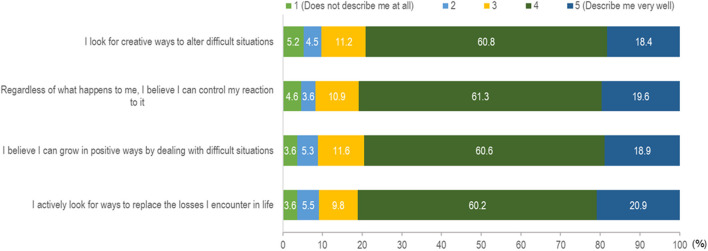
Responses for items in Brief Resilient Coping Scale (BRCS).

**Figure 3:**
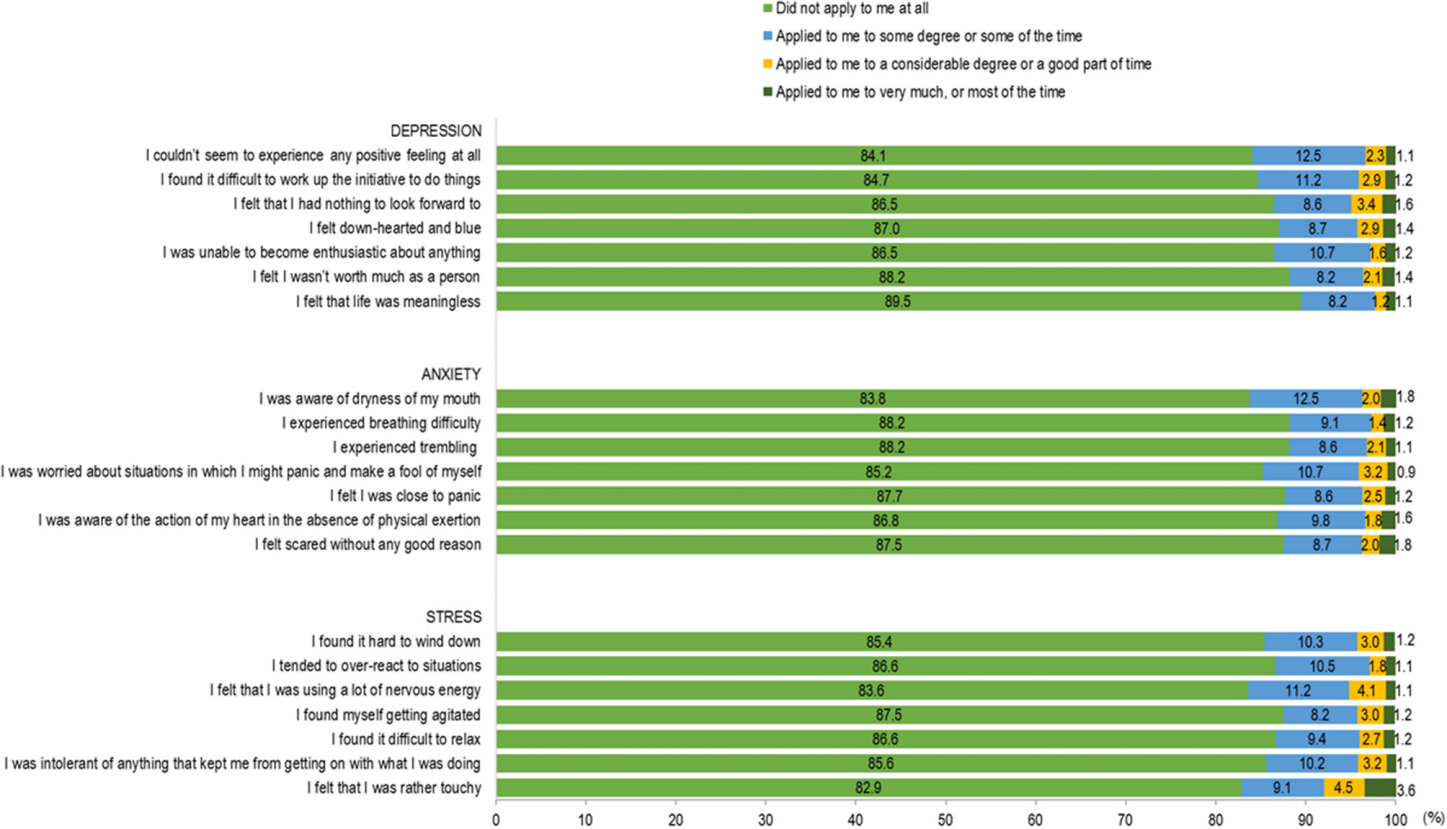
Responses for items in DASS-21.

**Figure 4:**
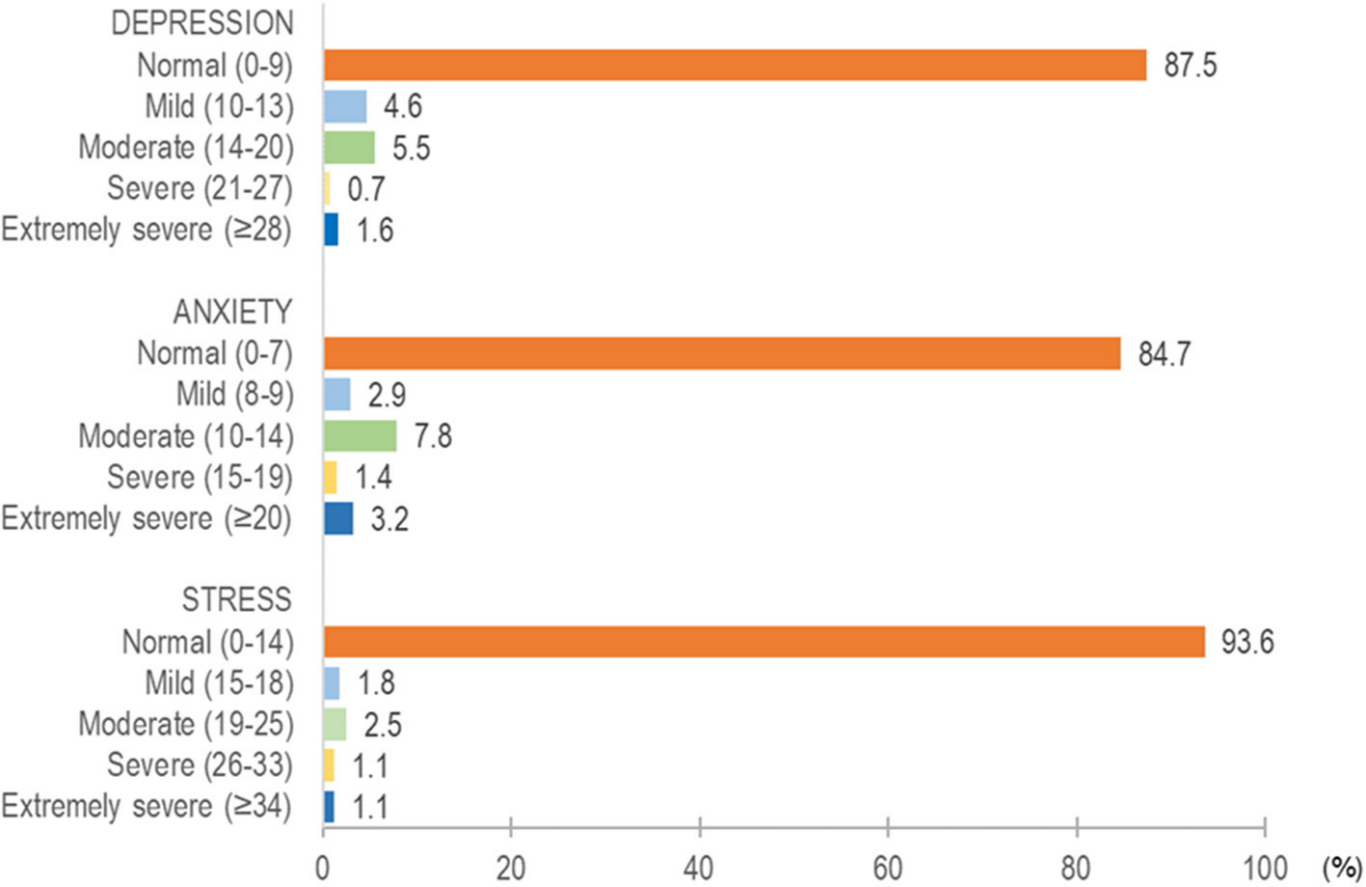
Severity rating of DASS-21.

The responses for the 4-item BRCS are shown in Figure 2. Of the 5-point Likert scale, the majority responded with 4 or 5 for all four items. As shown in Table 1, the majority (60.4%) reported median resilient coping (scores 14–16), followed by high resilient coping (scores 17–20) (22.1%). Figure 3 shows the distribution of responses in the DASS-21 items. Figure 4 shows the severity rating of DASS-21. A vast majority were found to have a normal score range in the three emotional states of depression, anxiety, and stress.

###  Psychological distress

Table 1 shows the proportion of participants with depression, anxiety, and stress symptoms. On the whole, depression, anxiety, and stress symptoms were reported in 12.5% (*n* = 70), 15.2% (*n* = 86), and 6.4% (*n* = 36) of participants, respectively. Multivariable logistic regression analysis showed that the parent–child conflict remains the strongest significant predictor for a higher level of depression (OR = 10.90, 95% CI 4.31–27.57), anxiety (OR = 11.92, 95% CI 5.05–28.14), and stress (OR = 4.79, 95% CI 1.41–16.33) symptoms.

Resilient coping was the second strongest predictor for depression and anxiety. There was an inverse association between resilient coping and depression and anxiety symptoms. Participants with a resilient coping score of 4–13 were associated with higher symptoms of depression than those with a resilient coping score of 17–20 (OR = 9.16, 95% CI 3.34–25.11). A resilient coping score of 4–13 was associated with higher anxiety symptoms than those with resilient coping scores of 17–20 (OR = 6.63, 95% CI 2.63–16.43).

Regarding lifestyle factors, participants who reported never or seldom carrying out the physical exercise in the past 3 months reported higher symptoms of depression (OR = 7.49, 95% CI 1.46–38.49). By demographics, females reported more severe symptoms of depression (OR = 3.11, 95% 1.35–7.19) than males. Young people from households with an income of MYR 2,000 and below have greater severity in symptoms of depression than those of households with an income between MYR 3,001 and 5,000 (OR = 5.93, 95% CI 2.23–15.75). Similarly, females reported greater severity in symptoms of anxiety (OR = 4.79, 95% 2.10–10.91) than males, and young people from a household income of MYR 2,000 and below reported greater severity in symptoms of anxiety than those of an income between MYR 3,001 and 5,000 (OR = 6.50, 95% CI 2.59–16.27). Young people who are employed also reported greater severity of anxiety symptoms than those who are unemployed (OR = 4.29, 95% CI 1.05–17.46). For stress symptoms, households with an income of MYR 2,000 and below reported higher stress levels than those with an income between MYR 3,001 and 5,000 (OR = 4.18, 95% CI 1.30–13.51).

## Discussion

The current study explored the responses and coping of Malaysian youth in low-income communities after the ease of the COVID-19 pandemic restrictions. Understanding the impact of the pandemic on these outcomes is critical for developing resources and interventions for families during and after the pandemic. This study sampled youth from a government settlement program and is known as the People's Housing Project (PPR). The PPR is an initiative by the Malaysian government to provide income earners under the Bottom 40% (B40) income groups to find a home and eradicate squatter areas in Malaysia. In this study, a high proportion had incomes of ≤ MYR 3,000 (81.4%), and a small proportion (18.5%) reported income between MYR 3,001 and 5,000. This implies that our study population closely represents the B40 group.

The results revealed that a minority of young people are adversely affected by the pandemic. Despite parent–child conflicts reported by a minority of young people in this study, it should not be underestimated as a healthy parent–child relationship is not only a key issue in family wellbeing, but it also represents whole-family functioning. Without the pandemic, parent–child conflict is a normal part of family life and often escalates during the teenage years. The biological and psychological changes in adolescence and youth may have a salient impact on parent–child relationships (25). Given that parent–adolescent conflict also has significant consequences for adolescent adaptation (26) and adolescents' behavioral and academic outcomes (27), intervention to promote positive parent–child relationships should be a part of public health priorities, particularly during social restrictions or crises.

In this study, parent–child conflict appears to be more prevalent among females and young people from the lowest income bracket in the underprivileged community. The finding of this study is in concordance with earlier studies before the COVID-19 pandemic that similarly found gender differences, with females showing an increase in parent–adolescent conflict intensity more than males (28, 29). Notably, females showed higher emotional expression (30) and higher sensitivity to stressors than their male counterparts (31), and this perhaps explains the higher parent–child conflicts in females. Interventions with parents and adolescents to prevent parent–child conflict should be used with an emphasis on young females. The association between lower income and poorer parent–adolescent conflict found in this study has also been reported in another study (32). Economic pressure was reported to have a significant impact on intra-family conflict (33). Our results indicate that households with more severe economic pressure may need help to prevent or mitigate family-related conflicts.

The current study found that there is generally a low prevalence of reported depression, anxiety, and stress symptoms among the sampled youth. Despite a low level of psychological distress, an important finding of this study is the strong association between parent–child conflict and psychological distress. The finding is congruent with a previous study that similarly reported a higher level of parent–child conflict was concurrently associated with greater depression symptoms among adolescents (34). Despite a low level of psychological stress and parent–child conflict, the findings of this study imply that it is still crucial to maintain a healthy parent–child relationship in shaping young people's mental health after the pandemic. Our findings provide key insights into the importance of positive parenting and the need to reduce conflicts within the family to sustain the mental health of young people even though economic activities and lifestyles have resumed normally.

The findings also revealed that females and those from low-income households are more likely to suffer higher psychological distress, hence providing insights into the vulnerable group of youth that should be targeted for counseling intervention to improve their mental wellbeing. Additionally, this study also found that young people who are employed were found to have higher anxiety symptoms than those who are unemployed or students. The COVID-19 pandemic has caused great concern regarding the overall mental health of employees worldwide. In particular, the pandemic has resulted in young adults facing an increased amount of psychological impairment linked to job insecurity and worsening career prospects (35). Our finding suggests that psychological interventions aimed at supporting mental health resilience among young people in work settings are essential in the post-COVID-19 period.

Of particular importance to highlight is that the majority of youth reported medium or high resilient coping, and resilient coping was the second strongest predictor for all three dimensions of psychological distress in this study. Resilience is an important predictor of the mental health of young people, primarily with respect to its positive indicator (36). Similarly, a study showed that strengthening resilience may lead to better mental wellbeing in young people (36). In this study, the majority of youth showed good and moderate resilient coping; this perhaps explains the low level of parent–youth conflicts and psychological distress reported in this study. It has been suggested that resilience-building programs for adolescents and youths are essential in increasing adaptability in the event of a future crisis or pandemic of infectious disease (37). Therefore, building and nurturing stress-resilient attitudes are essential to cultivating youth to be less vulnerable despite the experience of negative events.

There are some limitations to the current study that needs to be considered when interpreting the results. First, the cross-sectional design used could not infer a causal relationship. Second, the study sample represents a convenience sample of youth living in the PPR houses. The key disadvantage of convenience sampling is that the sample lacks clear generalizability. Furthermore, we only recruit youth from PPR houses in one state and federal territory in Malaysia; hence, the finding may be generalized to the entire population of low-income housing residents in Malaysia, a low-income community in Malaysia. Future research should include a more representative sample. Third, we cannot preclude the existence of recall errors and measurement bias when using retrospectively recalled information about lifestyle practices in the past 3 months. Finally, socially desirable responses to sensitive questions in this study may be one of the sources of bias leading to inaccurate self-reports and an erroneous study conclusion. Therefore, the findings of this study should be interpreted with caution.

## Conclusion

Young people from a low-income community reported low levels of parent–child conflict and psychological stress after the ease of the COVID-19 pandemic restrictions. Parent–child conflict is more prominent in females and young people from households with low-income earnings. Parent–child conflict was found to play a very prominent role in the increase in psychological distress. Family relationships are consequential for the psychological wellbeing of young people; therefore, it is necessary to pay attention to restoring family relationships during the post-pandemic period. The ability to cope with stress remains an important factor in reducing psychological distress; hence, young people should be provided support in building crisis resilience to enable them to cope adaptively with future stressful encounters. The study also identified the socially vulnerable youth groups in low-income communities, who should be the target for policies and services to overcome the aftermath impact of the COVID-19 pandemic and to better equip them in the event of future pandemics.

## Data availability statement

The raw data supporting the conclusions of this article will be made available by the authors, without undue reservation.

## Ethics statement

The studies involving human participants were reviewed and approved by the University of Malaya Research Ethics Committee (UM.TNC2/UMREC–1579). The patients/participants provided their written informed consent to participate in this study.

## Author contributions

LW, NF, SY, ZM, ZH, and YL contributed to the concept, design, and manuscript review. LW and HA contributed to the literature search, data acquisition, and statistical analysis. All authors contributed to the manuscript and approved the submitted version.

## Supplementary material

Appendix 1 Survey questionnaire.

Appendix 2 Parental Environment Questionnaire (PEQ) score by demographic characteristics.
