# Supplementary material for: COVID-19 responses and coping in young Malaysians from low-income families
Source: Front Psychiatry. 2023 May 15;14:1165023. doi: 10.3389/fpsyt.2023.1165023 (PMC10225688; doi:10.3389/fpsyt.2023.1165023)
Supplement: Appendix 1 — Survey questionnaire. [file Table_1.DOCX]

**HEALTHY PARENT-YOUTH RELATIONSHIPS DURING THE COVID-19 PANDEMIC: IDENTIFYING ISSUES AND NEEDS**

**Section A: General information**

| A1 | Age | ____ years old |
| --- | --- | --- |
| A2 | Gender | [ 1 ] Male  [ 2 ] Female |
| A3 | Occupation status | [ 1 ] Employed  [ 2 ] Unemployed  [ 3 ] Student |
| A4 | Monthly average household income (MYR) | [ 1 ] 1,000 and below  [ 2 ] 1,001 to 2,000  [ 3 ] 2,001 to 3,000  [ 4 ] 3,001 to 4,000  [ 5 ] 4,001 to 5,000 |
| A5 | Residence area | [ 1 ] Urban  [ 2 ] Sub-urban  [ 3 ] Rural |

**Section B: Lifestyle**

| B1 | In the past 3 months, what is your smoking status? | [ 1 ] Never smoke  [ 2 ] Former smoker  [ 3 ] Occasional smoker  [ 4 ] Regular smoker |
| --- | --- | --- |
| B2 | In the past 3 months, what is your alcohol use status? | [ 1 ] Never  [ 2 ] Former drinker  [ 3 ] Occasional drinker  [ 4 ] Regular drinker |
| B3 | In the past 3 months, how often do you exercise? | [ 1 ] Never  [ 2 ] Seldom  [ 3 ] Sometimes  [ 4 ] Often |
| B4 | In the past 3 months, how often do you practice healthy eating? | [ 1 ] Never  [ 2 ] Seldom  [ 3 ] Sometimes  [ 4 ] Often |
| B5 | In the past 3 months, how often do you have enough sleep in a week? | [ 1 ] Never  [ 2 ] Seldom  [ 3 ] Sometimes  [ 4 ] Often |

**Section C: Parent Environmental Questionnaire (PEQ)**

Now we would like to know more about your relationship with your parent

| C1 | My parent often loses her temper with me. | [ 1 ] Definitely true  [ 2 ] Probably true  [ 3 ] Probably false  [ 4 ] Definitely false |
| --- | --- | --- |
| C2 | There are often misunderstandings between my parent and myself. | [ 1 ] Definitely true  [ 2 ] Probably true  [ 3 ] Probably false  [ 4 ] Definitely false |
| C3 | My parent and I often get into arguments | [ 1 ] Definitely true  [ 2 ] Probably true  [ 3 ] Probably false  [ 4 ] Definitely false |
| C4 | My parent often criticizes me. | [ 1 ] Definitely true  [ 2 ] Probably true  [ 3 ] Probably false  [ 4 ] Definitely false |
| C5 | I often seem to anger or annoy my parent | [ 1 ] Definitely true  [ 2 ] Probably true  [ 3 ] Probably false  [ 4 ] Definitely false |
| C6 | My parent often hurts my feelings. | [ 1 ] Definitely true  [ 2 ] Probably true  [ 3 ] Probably false  [ 4 ] Definitely false |
| C7 | My parent often irritates me | [ 1 ] Definitely true  [ 2 ] Probably true  [ 3 ] Probably false  [ 4 ] Definitely false |
| C8 | My parent sometimes hits me in anger. | [ 1 ] Definitely true  [ 2 ] Probably true  [ 3 ] Probably false  [ 4 ] Definitely false |
| C9 | Once in a while I have been really scared of my parent. | [ 1 ] Definitely true  [ 2 ] Probably true  [ 3 ] Probably false  [ 4 ] Definitely false |
| C10 | Before I finish saying something, my parent often interrupts me | [ 1 ] Definitely true  [ 2 ] Probably true  [ 3 ] Probably false  [ 4 ] Definitely false |
| C11 | I treat others with more respect than I treat my parent. | [ 1 ] Definitely true  [ 2 ] Probably true  [ 3 ] Probably false  [ 4 ] Definitely false |
| C12 | My parent does not trust me to make my own decisions. | [ 1 ] Definitely true  [ 2 ] Probably true  [ 3 ] Probably false  [ 4 ] Definitely false |

**Section D: Brief Resilience and Coping Scale (BRCS)**

| Consider how well the following statements describe your behavior and actions on a scale from 1 to 5. | | | | | | |
| --- | --- | --- | --- | --- | --- | --- |
|  | | [ 1 ] [ 5 ]  Does not describe me at all Describes me very well | | | | |
| D1 | I look for creative ways to alter difficult situations. | [ 1 ] | [ 2 ] | [ 3 ] | [ 4 ] | [ 5 ] |
| D2 | Regardless of what happens to me, I believe I can control my reaction to it. | [ 1 ] | [ 2 ] | [ 3 ] | [ 4 ] | [ 5 ] |
| D3 | I believe I can grow in positive ways by dealing with difficult situations. | [ 1 ] | [ 2 ] | [ 3 ] | [ 4 ] | [ 5 ] |
| D4 | I actively look for ways to replace the losses I encounter in life. | [ 1 ] | [ 2 ] | [ 3 ] | [ 4 ] | [ 5 ] |

**Section E: DASS-21**

| Please read each statement and circle a number 0, 1, 2 or 3 which indicates how much the statement applied to you *over the past week*. There are no right or wrong answers. Do not spend too much time on any statement. | | | | | |
| --- | --- | --- | --- | --- | --- |
|  | | | | | |
|  |  | [ 0 ]  Did not apply to me at all | [ 1 ]  Applied to me to some degree, or some of the time | [ 2 ] Applied to me to a considerable degree, or a good part of time | [ 3 ]  Applied to me very much, or most of the time |
| E1 | I found it hard to wind down | [ 0 ] | [ 1 ] | [ 2 ] | [ 3 ] |
| E2 | I was aware of dryness of my mouth | [ 0 ] | [ 1 ] | [ 2 ] | [ 3 ] |
| E3 | I couldn't seem to experience any positive feeling at all | [ 0 ] | [ 1 ] | [ 2 ] | [ 3 ] |
| E4 | I experienced breathing difficulty (eg, excessively rapid breathing, breathlessness in the absence of physical exertion) | [ 0 ] | [ 1 ] | [ 2 ] | [ 3 ] |
| E5 | I found it difficult to work up the initiative to do things | [ 0 ] | [ 1 ] | [ 2 ] | [ 3 ] |
| E6 | I tended to over-react to situations | [ 0 ] | [ 1 ] | [ 2 ] | [ 3 ] |
| E7 | I experienced trembling (eg, in the hands) | [ 0 ] | [ 1 ] | [ 2 ] | [ 3 ] |
| E8 | I felt that I was using a lot of nervous energy | [ 0 ] | [ 1 ] | [ 2 ] | [ 3 ] |
| E9 | I was worried about situations in which I might panic and make a fool of myself | [ 0 ] | [ 1 ] | [ 2 ] | [ 3 ] |
| E10 | I felt that I had nothing to look forward to | [ 0 ] | [ 1 ] | [ 2 ] | [ 3 ] |
| E11 | I found myself getting agitated | [ 0 ] | [ 1 ] | [ 2 ] | [ 3 ] |
| E12 | I found it difficult to relax | [ 0 ] | [ 1 ] | [ 2 ] | [ 3 ] |
| E13 | I felt down-hearted and blue | [ 0 ] | [ 1 ] | [ 2 ] | [ 3 ] |
| E14 | I was intolerant of anything that kept me from getting on with what I was doing | [ 0 ] | [ 1 ] | [ 2 ] | [ 3 ] |
| E15 | I felt I was close to panic | [ 0 ] | [ 1 ] | [ 2 ] | [ 3 ] |
| E16 | I was unable to become enthusiastic about anything | [ 0 ] | [ 1 ] | [ 2 ] | [ 3 ] |
| E17 | I felt I wasn't worth much as a person | [ 0 ] | [ 1 ] | [ 2 ] | [ 3 ] |
| E18 | I felt that I was rather touchy | [ 0 ] | [ 1 ] | [ 2 ] | [ 3 ] |
| E19 | I was aware of the action of my heart in the absence of physical exertion (eg, sense of heart rate increase, heart missing a beat) | [ 0 ] | [ 1 ] | [ 2 ] | [ 3 ] |
| E20 | I felt scared without any good reason | [ 0 ] | [ 1 ] | [ 2 ] | [ 3 ] |
| E21 | I felt that life was meaningless | [ 0 ] | [ 1 ] | [ 2 ] | [ 3 ] |
