# Supplementary material for: COVID-19 responses and coping in young Malaysians from low-income families
Source: Front Psychiatry. 2023 May 15;14:1165023. doi: 10.3389/fpsyt.2023.1165023 (PMC10225688; doi:10.3389/fpsyt.2023.1165023)
Supplement: Appendix 2 — Parental Environment Questionnaire (PEQ) score by demographic characteristics. [file Table_2.DOCX]

Appendix 2 Socio-demographic characteristics factors associated with total Parental Environment Questionnaire (PEQ) score

|  | Frequency (%) | Univariable analysis | | |  | Multivariable analysis |
| --- | --- | --- | --- | --- | --- | --- |
|  |  | Total Parental Environment  Questionnaire (PEQ) Score | | |  | Total Parental Environment Questionnaire (PEQ) Score |
|  |  | Low score, High conflict  (12-47) | High score, Low conflict  (48) | p-value |  | Low score (12-47) *vs* High score (48)  OR (95% CI) |
| *Socio demographic characteristics* |  |  |  |  |  |  |
| Age group (years) |  |  |  |  |  |  |
| 18-21 | 391 (69.7) | 137 (35.0) | 254 (65.0) | 0.392 |  |  |
| 22-24 | 170 (30.3) | 66 (38.8) | 104 (61.2) |  |  |  |
| Gender |  |  |  |  |  |  |
| Male | 281 (50.1) | 76 (27.0) | 205 (73.0) | p<0.001 |  | Ref |
| Female | 280 (49.9) | 127 (45.4) | 153 (54.6) |  |  | 1.75 (1.19-2.57)** |
| Occupation status |  |  |  |  |  |  |
| Student | 354 (63.1) | 133 (37.6) | 221 (62.4) | 0.149 |  |  |
| Employed | 137 (24.4) | 52 (38.0) | 85 (62.0) |  |  |  |
| Unemployed | 70 (12.5) | 18 (25.7) | 52 (74.3) |  |  |  |
| Average monthly household income (MYR)^¶^ |  |  |  |  |  |  |
| 2000 and below | 100 (17.8) | 75 (75.0) | 25 (25.0) | p<0.001 |  | 4.39 (2.40-8.03)*** |
| 2001-3000 | 357 (63.6) | 85 (23.8) | 272 (76.2) |  |  | 0.51 (0.32-0.82)** |
| 3001-5000 | 104 (18.5) | 43 (41.3) | 61 (58.7) |  |  | Ref |
| Residence area |  |  |  |  |  |  |
| Urban | 484 (86.3) | 169 (34.9) | 315 (65.1) | 0.126 |  |  |
| Sub-urban | 77 (13.7) | 34 (44.2) | 43 (55.8) |  |  |  |

**p<0.01, ***p<0.001

Hosmer–Lemeshow test, chi-square: 12.653, p-value: 0.013; Nagelkerke R^2^: 0.217

^¶^ 1USD = 4.41 MYR
